# 

**Published:** 1906-12-15

**Authors:** 


### CONTENTS.

Event and Comment - - - - - - - - - - - 595

A Plea for the Adoption of Oral Prophylaxis,

By Grace P. Rogers, D.D.S. 597

Little Helps, - - - - - By George Zederbaum, D.D.S. 618

Odontalgia, - - - - - By Wm. D. McFadden, D.D.S. 620

The Proper Light and Arrangement of the Dental Operating Rooms,
By George B. Sanford, D.D.S. 622

With Our Editors. - - - - - - - - - - - 623

Indents - - - - - - - - - - - - 630

Announcements - - - - - - - - - - - 636

